# A multi-stakeholder perspective on asthma care in Canada: findings from a mixed methods needs assessment in the treatment and management of asthma in adults

**DOI:** 10.1186/s13223-018-0261-x

**Published:** 2018-09-10

**Authors:** Suzanne Murray, Sara Labbé, Alan Kaplan, Kristine Petrasko, Susan Waserman

**Affiliations:** 1 AXDEV Group Inc., 210-8, Place du Commerce, Brossard, QC J4W 3H2 Canada; 2 Department of Family and Community Medicine, University of Toronto, 500 University Ave, Toronto, ON M5G 1V7 Canada; 3 Winnipeg Regional Health Authority, Winnipeg, MB R2R 2S8 Canada; 4 Division of Clinical Immunology and Allergy, McMaster University, 1280 Main St West, HSC 3V49, Hamilton, ON L8S 4K1 Canada

**Keywords:** Needs assessment, Asthma, Continuing medical education, Mixed-methods, Clinical challenges, Clinical practice, Guidelines

## Abstract

**Background:**

Although several aspects of asthma care have been identified as being sub-optimal in Canada, such as patient education, practice guideline adoption, and access to care, there remains a need to determine the extent to which these gaps remain, so as to investigate their underlying causes, and potential solutions.

**Methods:**

An ethics-approved mixed methods educational needs assessment was conducted in four Canadian provinces (Alberta, British Columbia, Ontario, and Quebec), combining a qualitative phase (45-min semi-structured interviews with community-based healthcare providers and key stakeholders) and a quantitative phase (15-min survey, healthcare providers only).

**Results:**

A total of 234 participants were included in the study, 44 in semi-structured interviews and 190 in the online survey. Five clinical areas were reported to be suboptimal by multiple categories of participants, and specific causes were identified for each. These areas included: Integration of guidelines into clinical practice, use of spirometry, individualisation of asthma devices to patient needs, emphasis on patient adherence and self-management, and clarity regarding roles and responsibilities of different members of the asthma healthcare team. Common causes for gaps in all these areas included suboptimal knowledge amongst healthcare providers, differing perceptions on the importance of certain interventions, and inadequate communication between healthcare providers.

**Conclusions:**

This study provides a better understanding of the specific causes underlying common gaps and challenges in asthma care in Canada. This information can inform future continuing medical education, and help providers in community settings obtain access to adequate materials, resources, and training to support optimal care of adult patients with asthma.

## Background

The prevalence of asthma in Canadian adults has more than tripled over the last three decades, but remains stable since 2004 with a current prevalence of 8.1%, affecting an estimated 2.4 million Canadians [1]. Several aspects of asthma care and management in Canada have previously been identified to be sub-optimal, specifically in relation to patient education, adoption of practice guidelines by healthcare providers, and issues specific to the structure and regulations of the different provincial healthcare systems [2–4]. A 2006 Canadian survey of patients and physicians demonstrated a lack of patient understanding of controlled vs. uncontrolled asthma, and inadequate use of Canadian asthma guidelines by physicians [4]. Poor inhaler technique had also been identified as an important contributor to decreased patient adherence, and sub-optimal asthma control [3]. More recently, a questionnaire was developed to assess family physicians’ integration of the Canadian asthma guidelines into practice, and revealed that, despite guideline recommendations, there was a significant lack of education provided to patients [2]. From a systems perspective, lack of access to spirometry testing has been reported as an important barrier to accurate diagnosis and monitoring of asthma [5, 6].

Direct and indirect impacts of asthma have been well documented in the literature for the past two decades, and include significantly reduced patient quality of life, work absenteeism, and substantial healthcare and productivity costs [7–9]. In addition, uncontrolled asthma is responsible for the death of over 200 adults Canadians each year (228 in 2009 [10]), highlighting the importance of investigating potential contributors to sub-optimal care in asthma.

Although multiple barriers have been identified in Canada in the last 20 years, it is not known to what extent these barriers currently remain. In addition, the precise causes of these barriers are often not investigated, limiting the effectiveness of potentially corrective interventions. A previous international study, conducted by a group of researchers that included co-authors SM and SW, compared the state of asthma care in 4 countries (Canada, France, Germany and the United Kingdom) [11] and identified four main challenges: (1) awareness and understanding of asthma including severe asthma, (2) diagnosis of severe asthma, (3) new treatments and personalized medicine, (4) referral process and collaboration between primary care and specialty care.

### Study rationale and objectives

To determine the extent to which previously identified gaps and barriers in adult asthma care still exist, as well as their underlying causes and potentially new challenges, an in-depth mixed methods educational needs assessment was conducted. An educational needs assessment consists of a systematic investigation of how “what is” differs from “what should be”, in order to identify the educational needs of a defined population [12]. This study collected the perspectives of community-based (i.e. non-academic) healthcare providers and other key stakeholders from the four largest provinces of Canada (Alberta, British Columbia, Ontario, and Quebec). The objectives of this study were to identify the challenges faced within the Canadian healthcare system, their causes, and to recommend interventions that could bridge these care gaps and ultimately improve patients’ asthma care.

## Methods

### Overview of the mixed methods approach

This Canadian needs assessment used a mixed methods approach which consisted of collecting data through two consecutive phases; a qualitative phase followed by a quantitative phase. The qualitative phase consisted of semi-structured interviews. The qualitative findings were used to design the quantitative online survey. Triangulation of data sources (different categories of participants) and data collection methods (interviews, survey) were used to increase validity of findings [13]. A mixed methods study design allows the benefit of two types of data collection: the depth, breadth and exploratory nature of qualitative methodology, and the precision and analytic power of quantitative methodology [14].

### Recruitment

Potential participants were identified mainly through professional listings purchased from independent (not pharmaceutical industry-related) organization in compliance with the ESOMAR/ICC International Code on Market, Opinion and Social Research and Data Analytics [15]. Other recruitment methods included snowball sampling, which consists of asking initial participants to refer potential participants from their own social network [16]. Invitations to participate in the telephone interviews (phase 1) were sent via email, which included a link to a secure website that provided study details, screening questions, and an informed consent agreement. Eligible individuals who consented to participate were then redirected to an availability form to schedule their interviews. Recruitment for telephone interviews closed once targeted numbers of participants were reached. Invitations to participate in the online survey (phase 2) were then sent with the same process, and eligible individuals were redirected to the survey.

Eligibility criteria for healthcare providers included (1) in active practice for a minimum of 3 years, (2) a primary role as a clinician (not research or teaching) from an eligible profession/specialty (see below) in a community setting (non-academic), (3) in either Alberta, British Columbia, Ontario or Quebec, and (4) a minimum caseload of 20 adults patients with asthma per month. Eligible professions were: (1) allergists/clinical immunologists, respirologists, pneumologists (grouped together as “specialists”); (2) general practitioners, family physicians (GP/FP); (3) Community Pharmacists; (4) Nurses; (5) Certified Respiratory Educators (CRE). Nurses and pharmacists who self-reported having a Respiratory Educator certification were classified as CRE. Sample size for each of these groups were determined based on their population size and professional involvement in asthma care in the community setting. Therefore, the target sample for pharmacists and family physicians are higher than for other professionals such as specialists, nurses with asthma patients and CRE.

Administrators were required to have at least 2 years experience in the administration or management of a community clinical institution. Patient advocates were required to be involved with a recognized patient advocacy group at the national or provincial level. Payers were required to be involved or to have been recently involved with a private or public insurer. For the purpose of this study, “policy influencers” were defined as individuals who have expertise in, and influence on health policy, and/or are experts in the field of asthma in Canada.

### Data collection

A review of the literature on existing gaps, barriers and challenges in adult asthma care was initially conducted to identify the main areas to be discussed with interview participants in the qualitative phase of the study. These areas of exploration were discussed with clinical experts (co-authors AK, KP and SW) and researchers in the field of medical education (including co-authors SM, SL). The final areas were used to design the interview guide. For each area, open-ended, non-directive questions were designed to collect in-depth data about challenges and barriers to optimal asthma care from participants. The 45-min telephone interviews were conducted by trained interviewers in educational research (including co-authors SM and SL) and conducted in the two official languages of Canada (English and French). Interviews were audio-recorded (with each participant’s consent) and transcribed for analysis.

The online survey questions were designed based on the themes that emerged from the qualitative analysis. Survey was completed by healthcare providers only, as they were the primary target for the continuing medical/health education this study aimed to inform. The 15 min survey consisted of six sections: (1) self-assessment of level of knowledge in relation to specific components of asthma guidelines; (2) self-assessment of confidence; (3) skills in relation to specific tasks that should be performed in the treatment and management of asthma patients; (4) perceived importance of these specific tasks in the delivery of optimal asthma care; (5) agreement level with statements related to asthma care; and (6) ranking or perceived importance of significant barriers to the provision of optimal asthma care. Response formats of survey questions included multiple nominal choices, Likert-type scales and visual-analogue scales, all of which were used in previous needs assessments [17].

The 15-min online survey was designed in English and translated into French. To increase validity of data, the survey questions were adapted to the specific roles and responsibilities of the participants; for example, some questions were asked only to nurses, to pharmacists, or to healthcare providers licensed to prescribe. For Sections 1, 2 and 3, participants were asked to self-report their knowledge, confidence and skills in relation to their professional role.

### Analysis plan

Transcribed interviews (qualitative phase) were analysed using a four-step approach derived from thematic analysis [18] and directed content analysis [19] using NVivo qualitative data analysis software (QSR International Pty Ltd, Version 7, 2006). These steps consisted of (1) developing the coding tree with pre-determined codes based on the initial areas of investigation and study objectives; (2) coding the transcripts according to the pre-determined codes; (3) refining the coding tree based on data that could not be coded with the predetermined codes; and (4) identification of themes that emerged the most frequently across and within different sources.

Data from the online survey (quantitative phase) was analysed using IBM SPSS 22.0 software (IBM Corporation, Armonk, NY). Data was analysed using frequencies, cross tabulations, means and Chi square. Post-hoc tests were performed when Chi square was significant (p < 0.05). To identify potential educational gaps, knowledge and skill answers were recoded from 5-point Likert-type scale into dichotomous variables, either as a 1–3 response (“low” to “acceptable”), or a 4–5 response (“optimal”).

Main gaps, barriers and challenges to optimal asthma care as well as their causes such as sub-optimal knowledge, skills, or confidence were identified using the triangulation of data sources (professions) and methodologies (qualitative and quantitative). Data was interpreted by clinical experts (co-authors AK, KP, SW) and educational experts in the field of health care (co-authors SM and SL).

## Results

### Sample characteristics

The study sample included a total of 233 participants. Semi-structured telephone interviews were conducted with 43 participants (37 health-care providers and 6 non-healthcare providers) and the online survey was completed by 190 health-care providers. Table 1 presents the sample, grouped by professions for each phase. The majority of participants (84%) had more than 10 years of practice and nearly half of healthcare providers (46%) had 50 or more adult asthma patients a month.

**Table 1:** Description of the study sample

Profession	Phase I: qualitative (interview)	Phase II: quantitative (online survey)	Total
General practitioners/family physicians	8	79	*87*
Specialists^a^	8	18	*26*
Nurses	8	18	*26*
Pharmacists	5	54	*59*
Certified Respiratory Educators (CRE)^b^	8	21	*29*
*Non-healthcare providers (sources of triangulation)*			
Admins/payers/policy influencers	4	–	*4*
Patient advocates	2	–	*2*
*Total*	*43*	*190*	*233*

^a^Including allergists/clinical immunologists, respirologists and internal medicine specialists

^b^Including nurses and pharmacists who have obtained a Respiratory Educator certification

The following sections present challenges in adult asthma care, and their underlying causes, as reported by participants in the qualitative and quantitative phases. This manuscript will focus on five clinical areas that were substantively reported to be problematic by multiple categories of participants: (1) integration of guidelines into clinical practice; (2) use of spirometry; (3) individualisation of asthma devices to patient needs; (4) promotion of patient adherence and self-management; and (5) definition and sharing of roles and responsibilities by asthma healthcare professionals.

#### Challenges with integration of asthma guidelines into clinical practice

An inadequate level of knowledge was reported by participants regarding the Canadian Thoracic Society (CTS) and the Global Initiative for Asthma (GINA) guidelines for adult asthma care (see Table 2). Overall, knowledge was reported to be lower for the GINA guidelines with a total of 77% participants reporting sub-optimal knowledge as compared to 64% for the CTS guidelines. Nurses were the professional sub-group reporting the highest gaps in knowledge for these two guidelines, *in relation to what it should be, given their professional role*: 83% for the CTS guidelines (significant differences between professions, p < 0.001) and 94% for the GINA guidelines. More than half of CREs and GP/FPs also reported knowledge gaps in these two guidelines: with CREs reporting less knowledge of the CTS guidelines (67% CTS, vs. 57% for GINA) whereas a higher proportion of GP/FP reported less knowledge of GINA (77% GINA vs. 52% CTS). From one-third to one quarter of specialists reported a gap for each of the guidelines (28% for CTS; 33% for GINA). Interviewed participants explicitly expressed the need to improve overall knowledge of guidelines for the asthma care community, and to enhance implementation in practice:

**Table 2:** Sub-optimal knowledge reported by healthcare providers

Knowledge area	% (n) of participants who reported sub-optimal knowledge in relation to what it should be, given their professional role^a^						
	GP/FPs. (n = 79)	SPE. (n = 18)	CRE. (n = 21)	Nurses (n = 18)	Pharm. (n = 54)	Total (n = 190)	Significant differences^b^
Canadian Thoracic Society (CTS) guidelines	52%^c^ (n = 41)	28%^c^ (n = 5)	67% (n = 14)	83% (n = 15)	87%^c^ (n = 47)	64% (n = 122)	p < 0.001
Global Initiative for Asthma (GINA) guidelines	77% (n = 61)	33% (n = 6)	57% (n = 12)	94% (n = 17)	93% (n = 50)	77% (n = 146)	NV
Indicators to request or conduct a spirometry test	33%^c^ (n = 26)	22% (n = 4)	38% (n = 8)	44% (n = 8)	89%^c^ (n = 48)	50% (n = 94)	p < 0.001
Respective responsibilities of healthcare team members regarding patient education, in my practice setting	27% (n = 21)	22% (n = 4)	24% (n = 5)	44% (n = 8)	41% (n = 22)	32% (n = 60)	NS

*GP* general practitioner, *FP* family physician, *SPE* specialist, *CRE* Certified Respiratory Educator, *Pharm* community pharmacist, *NS* not significant, *NV* Chi square not valid due to distribution

^a^Self-reported 1–3 on a 5-pt scale, where 1 = low, given my professional role 3 = acceptable, but could be improved, given my professional role and 5 = optimal, given my professional role

^b^Significant differences between professions using Chi square (p < 0.05)

^c^Post hoc test indicated for statistical difference

*“I would say that I’ve worked in six offices in the last ten years, same thing, no*-*one actually has a consistent evidence*-*based way of assessing. And yet the checklist is there. (…) The question is, who does this?”*

-FP/GP

Discrepancies between these two guidelines were reported by participants of this study. Half of participants (49%) agreed with the statement that “there are discrepancies between the Canadian guidelines and the international guidelines which create confusion on what to do in practice” (see Table 3). This proportion of agreement reached 72% among nurses and SPE compared to 43% of CRE and 41% of GP/FPs (p = 0.035). Discrepancies between guidelines, especially for criteria determining asthma control, were also explicitly reported by interviewed participants, and participants reported a preference for the GINA guidelines over the CTS:

**Table 3:** Participants’ level of agreement with statements on asthma care

Level of agreement with…	% (n) of participants who reported agreement with the statement^a^						
	GP/FPs. (n = 79)	SPE. (n = 18)	CRE. (n = 21)	Nurses (n = 18)	Pharm. (n = 54)	Total (n = 190)	Significant differences^b^
I believe there are discrepancies between the Canadian guidelines and the international guidelines which creates confusion of what to do in practice	41% (n = 32)	72% (n = 13)	43% (n = 9)	72% (n = 13)	50% (n = 27)	49% (n = 94)	p = 0.035
Asthma spirometry test is not necessary to diagnose asthma	43%^c^ (n = 34)	44% (n = 8)	14% (n = 3)	17% (n = 3)	17% (n = 9)	30% (n = 57)	p = 0.002
Asthma can be diagnosed based on patient history, and response to a medication trial	75% (n = 59)	72% (n = 13)	71% (n = 15)	50% (n = 9)	63% (n = 34)	68% (n = 130)	NS
Most patients with asthma do not proactively help themselves	56% (n = 44)	67% (n = 12)	48% (n = 10)	33% (n = 6)	61% (n = 33)	55% (n = 105)	NS
Managing adult patients with asthma is time-consuming and frustrating	35% (n = 28)	72% (n = 13)	33% (n = 7)	39% (n = 7)	39% (n = 21)	40% (n = 76)	NS
I suspect there is more I should be doing in the care of patients with asthma	72% (n = 57)	67% (n = 12)	81% (n = 17)	89% (n = 16)	87% (n = 47)	78% (n = 149)	NV

*GP* general practitioner, *FP* family physician, *SPE* specialist, *CRE* Certified Respiratory Educator, *Pharm* community pharmacist, *NS* not significant, *NV* Chi square not valid due to distribution

^a^Participants were asked to indicate their level of agreement with the following statements. Data are the % of participants that selected 3 or 4 on a 4-pt scale (1 = completely disagree, 2 = slightly disagree, 3 = slightly agree, 4 = completely agree)

^b^Significant differences between professions using Chi square (p < 0.05)

^c^Post hoc test indicated for statistical difference

*“I find that the Canadian guidelines are not, I guess, tight enough, or they’re too relaxed in determining control. So unlike the GINA guidelines where they say zero symptoms not using your beta*-*2 at all. The Canadian guidelines, I mean they’re allowing up to four doses of your beta*-*2 in a week. And I just find that just that, that will mix up our doctors.”*

-CRE

*“The GINA guidelines, the Global Initiative for Asthma is* [sic] *up to date, 2016, but our Canadian guidelines are not, 2012. And they’re not consistent therefore. So, there’s contradiction sometimes or there’s gaps in what the CTS guidelines talk about. So, it’s another confusing picture. I think the GINA guidelines … should be exercised, they’re very complete and they’re easier to use*.”

-FP/GP

#### Challenges with the use of spirometry for diagnosis and management

When asked about their knowledge of when to request spirometry, an insufficient level of knowledge was reported by nurses (44%), CRE (38%), and GP/FPs (33%).

Almost half of GP/FPs (43%) and specialists (44%) agreed with the statement that “Spirometry test is not necessary to diagnose asthma” as compared to 17% of nurses and 14% of CRE (p = 0.002, see Table 3). In addition, three-quarters of CRE, GP/FPs and SPE (71, 75 and 72% respectively) agreed that asthma can be diagnosed based on patient history and response to a trial of medication. Confirming an asthma diagnosis was also found not to be necessary at all, or necessary only in specific cases by nearly half of GP/FPs (46%) (see Table 4).

**Table 4:** Participants’ perceived importance of doing specific tasks in their current clinical practice

Perceived importance of…	% (n) of participants who reported the task as necessary						
	GP/FPs. (n = 79)	SPE. (n = 18)	CRE. (n = 21)	Nurses (n = 18)	Pharm. (n = 54)	Total (n = 190)	Significant differences^b^
Confirm diagnosis prior to initiating treatment	54% (n = 43)	94% (n = 17)	Not asked	Not asked	Not asked	62% (n = 60)	NV
Select the type of device based on my patient’s preferences	73% (n = 58)	94% (n = 17)	67% (n = 14)	61% (n = 11)	83% (n = 45)	76% (n = 145)	NV
Assess proper use of device with a demonstration	76% (n = 60)	78% (n = 14)	81% (n = 17)	61% (n = 11)	93% (n = 50)	80% (n = 152)	NV

*GP* general practitioner, *FP* family physician, *SPE* specialist, *CRE* Certified Respiratory Educator, *Pharm* community pharmacist, *NV* Chi square not valid due to distribution

^a^Selected 4 or 5 on a 5-pt scale (1 = Not necessary at all, 3 = necessary only in specific cases, and 5 = always necessary)

^b^Significant differences between professions using Chi square (p < 0.05)

This perception of spirometry not being necessary to diagnose asthma was also an important theme that emerged from the semi-structured interviews:

*“I only diagnose by history and physical. If I think there’s some COPD or some other chronic respiratory illness then they may go for spirometry* ***but I tend to think of spirometry more for COPD*** *than asthma and so for* ***asthma patients most of them are just diagnosed by history and physical and I only use the spirometry if I want to rule something else out*** *and that’s it, and then* ***I give them a trial of medication and see if they’re better with the medication.”***

-FP/GP

Spirometry was also reported to be underused to monitor asthma control. As summarized in Table 5, over three quarters of CREs (76%) and half of GP/FPs (56%) and nurses (50%) reported using spirometry for monitoring asthma control either never or only at the first consultation, compared to 17% of specialists. CREs were also the profession reporting a lesser use of spirometry for monitoring asthma symptoms and exacerbations (62% never doing it or only on first consultation), followed by nurses (56%), GP/FPs (54%), and specialists (28%).

**Table 5:** Participants reporting of the frequency they are doing specific tasks in their current clinical practice

Task	% (n) of participants who report never doing the task or only in the first consultation with their patients^a^						
	GP/FPs. (n = 79)	SPE. (n = 18)	CRE. (n = 21)	Nurses (n = 18)	Pharm. (n = 53)	Total (n = 189)	Significant differences^b^
Assess asthma control with spirometry	56% (n = 44)	17% (n = 3)	76% (n = 16)	50% (n = 9)	93% (n = 49)	64% (n = 121)	NV
Assess asthma symptoms and exacerbations with spirometry	54% (n = 43)	28% (n = 5)	62% (n = 13)	56% (n = 10)	94% (n = 50)	64% (n = 121)	NV

*GP* general practitioner, *FP* family physician, *SPE* specialist, *CRE* Certified Respiratory Educator, *Pharm* community pharmacist, *NV* Chi square not valid due to distribution

^a^Other nominal answer choices provided were “In most of my patients’ consultations” and “Systematically in each of my patients’ consultations”

^b^Significant differences between professions using Chi square (p < 0.05)

**Fig. 1:**
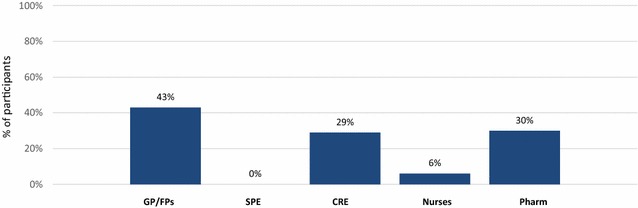
Perceived lack of access to spirometry selected by GP/FPs as an important barrier to provide optimal asthma care. *GP* general practitioner, *FP* family physician, *SPE* specialist, *CRE* Certified Respiratory Educator, *Pharm* Community pharmacist. Participants were asked to select the five most important barriers, among a list of 14, to provide optimal care to patients with asthma. Data are percentage of participant that selected *Lack of access to spirometry in my practice setting* as a barrier among a list of 14

Among GP/FPs, 43% selected “lack of access to spirometry in their practice setting” as an important barrier to providing optimal asthma care whereas 0% of SPE selected this item as an important barrier (p = 0.001, see Fig. 1). The need to have better access to spirometry for community family physicians was frequently mentioned by interviewed participants:

“***The spirometry is not universally available in the community****. What we do in our office is all of our tests are performed by respiratory therapists, which is quite expensive* […] *I have sympathy for the primary care physicians that often they don’t have the availability of the tools such as spirometry to help them.”*

-Specialist

#### Challenges with the individualisation of devices to patient needs

Over a third of nurses perceived that individualising the type of device based on their patient’s preferences (39%) and assessing/demonstrating its proper use (also 39%) to not be necessary or to be necessary only in specific cases (see Table 4).

As shown in Table 6, sub-optimal skills selecting the device best adapted to a given patient were also reported in a higher proportion by nurses (61%) as compared to other participants (p = 0.029). Pharmacists had the lowest proportion of participants reporting a skill gap related to this task (20%).

**Table 6:** Sub-optimal skills reported by healthcare providers

Skill	% (n) of participants who reported sub-optimal skills in relation to what it should be, given their professional role^a^						
	GP/FPs. (n = 79)	SPE. (n = 18)	CRE. (n = 21)	Nurses (n = 18)	Pharm. (n = 54)	Total (n = 190)	Significant differences^b^
Selecting/recommending the most adapted device to a given patient	33% (n = 26)	28% (n = 5)	29% (n = 6)	61%^c^ (n = 11)	20% (n = 11)	31% (n = 59)	p = 0.029
Promoting self-management	17% (n = 13)	6% (n = 1)	14% (n = 3)	39% (n = 7)	28% (n = 15)	21% (n = 39)	NV

*GP* general practitioner, *FP* family physician, *SPE* specialist, *CRE* Certified Respiratory Educator, *Pharm* community pharmacist, *NV* Chi square not valid due to distribution

^a^Self-reported 1 to 3 on a 5-pt scale, with 1 = low, given my professional role 3 = acceptable, but could be improved, given my professional role and 5 = optimal, given my professional role

^b^Significant differences between professions using Chi square (p < 0.05)

^c^Post hoc test indicated for statistical difference

Increasing variety of available type of devices was mentioned by interviewed participants as a contributor to the difficulty in selecting the most suitable device to the patient needs:

“*There are about fifteen kinds* […] *that’s a lot… There’s at least ten devices to use the inhaler, and it doesn’t work with all the patients: people are more familiar with one or the other, and it needs to be tested*”.

-Specialist

#### Challenges with promotion of patient adherence and self-management

There was a perception from interviewed participants that lack of patient adherence is often explained by an overall disengagement of the patients from their condition, generating frustration within the health-care team. As detailed in Table 3, more than half of all participants (55%) agreed with the statement that “most patients with asthma do not proactively help themselves”. Specialists agreed with this statement (67%), followed by pharmacists (61%) and GP/FPs (56%). In addition, a large majority of specialists (72%) agreed that managing adult patients with asthma is time-consuming and frustrating. Other professions also agreed with this statement although to a lesser extent (see Table 3).

**Fig. 2:**
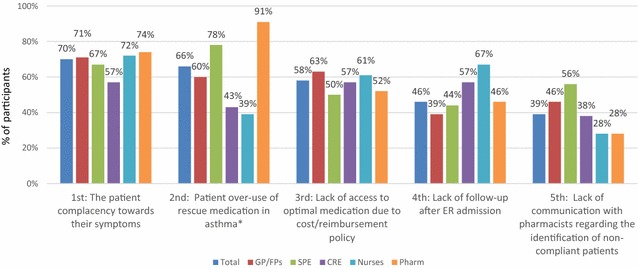
Top 5 most often selected barriers to providing optimal care. *GP* general practitioner, *FP* family physician, *SPE* specialist, *CRE* Certified Respiratory Educator, *Pharm* Community pharmacist. Participants were asked to select the five most important barriers, among a list of 14, to providing optimal care to patients with asthma. Barriers presented are the top five most selected in total. Data are % of participants who selected that barrier

Patient complacency toward their symptoms was the most often identified barrier to providing optimal care, selected by 70% of participants. As summarized in Fig. 2, pharmacists (74%) selected this most often, followed by nurses (72%) and GP/FPs (71%). In addition, patient overuse of rescue medication, a specific type of non-adherence, was the second being selected by 66% of participants, and especially by pharmacists (91%) (p = 0.001). The sense of frustration of healthcare providers when treating and managing asthma was reported to be especially high when providing care to young, busy adults:

“*I would say probably a young healthy otherwise healthy asthmatic, especially male. I have quite a few patients between the ages of like twenty to thirty. They are hard to treat because of compliance and trying to get them to take their asthma seriously. The problem is compliance and getting them to book appointments if they work, as they have to take time off work.* “

-CRE

**Fig. 3:**
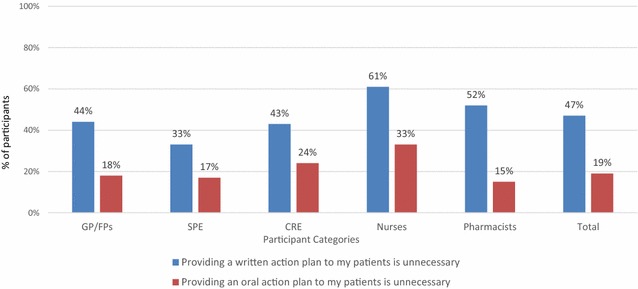
Perceived importance of providing an oral versus a written action plan. *GP* general practitioner, *FP* family physician, *SPE* specialist, *CRE* Certified Respiratory Educator, *Pharm* community pharmacist. Question asked: Please indicate how necessary, in your professional role, are the following items in your practice with adult patients suffering from asthma. Scale: 1 = Not necessary at all, 3 = Only necessary in specific cases and 5 = Always necessary. Data are the percentage of participants that selected 1, 2 or 3

As illustrated in Fig. 3, 47% of participants reported that providing a written action plan to patients is not at all necessary or only in certain cases. Specifically, near half of CRE (43%), GP/FPs (44%), and half of pharmacists (52%) perceived that providing a written plan as not necessary. Providing an oral action plan was perceived as more important than a written plan, although, 19% of participants reported that providing oral plan was not necessary at all, or only in specific cases.

A large majority of participants agreed they should be doing more when caring for patient with asthma (78%). As summarized in Table 3, the level of agreement reached 89% among nurses and 87% among pharmacists.

The overall lack of perceived importance by the healthcare team of providing the patient with a written plan, was also a main theme identified by multiple type of participants during the semi-structured interviews:

*“I do not* [use a written action plan]. *Because I show them. So, I will draw something on my examination paper on my room and I’ll take a couple of puffs up to my fake inhaler and I’ll do it that way.* ***But I do not give people a written plan****.”*

-FP/GP

The following quote by an asthma clinic administrator illustrates how important providing a written plan should be to promote patient compliance:

*“That* [written] *asthma action plan is the bee’s knees to me.* ***That’s one thing that just lets the patient know when and why they would need to be concerned, and how they should be treating their asthma***. […] *If they’ve never gone through it* […] *how would they know? They’re just treating themselves as they think they should be.”*

-Asthma clinic administrator

#### Sub-optimal sharing of roles and responsibilities among health-care team, especially regarding patient education

As shown in Table 2, almost half of nurses and pharmacists reported knowledge gaps of the roles of respective healthcare team members (44 and 41%). The lack of communication with pharmacists regarding the identification of non-compliant patients was among the top five barriers the most often selected among a list of 14 and was selected by 46% of GP/FPs and 56% of specialists (see Fig. 2).

This lack of clarity about team members’ roles and responsibilities emerged as an important theme from the semi-structured interviews:

*“I think asthma care could be optimized if there was more education around what the specific role of each health professional (doctor, pharmacist) was in regards to asthma care.* ***I am not confident that all physicians are monitoring their asthma patients using spirometry/peak flow, and if pharmacists were aware of this gap in treatment they may make more of an effort to fill the gap*** *.”*

-Pharmacist

Participants in semi-structured interviews expressed the need to improve the clarity of roles and responsibilities of healthcare professionals, especially pharmacists, in the management of adult patients with asthma, with the objective of providing clear information to patient:

*“Different information coming from a physician/pharmacist and then information coming through me, if it’s not the same. That would be probably one of the hardest, the biggest barriers to get over directly with a patient.”*

-CRE

“*What works not so well, consistency between healthcare professionals. The patients are often getting mixed messages. I had mentioned the problem with a pharmacist earlier. That would be one example*.”

-Specialist

## Discussion

This study provides evidence of clinical challenges experienced by healthcare providers in Canada in five areas related to treatment and management of patients with asthma in community settings. Challenges and their causes were identified in five specific areas: asthma practice guidelines, use of spirometry, individualisation of devices, patient adherence, and sharing of roles among the multidisciplinary team.

A main finding of this report is related to the poor integration of asthma guidelines into practice. This finding could be explained by a lack of exposure to guidelines among health care teams, as well as the lack of access to spirometry in primary care settings, combined with the widespread belief that spirometry is not necessary to diagnose or monitor asthma.

The resulting general underuse of spirometry in primary care settings, and its relationship to uncertainty surrounding asthma diagnoses, emerged as another important issue in this study, as it did in others conducted in the U.S., Asia, and Europe [20]. Health care teams simply may not perceive the value of incorporating these guidelines into practice, nor the consequences of failing to do so.

One of these consequences is a contribution to the over-diagnosis of asthma in Canada [21, 22]. For example, a recent Canadian study conducted among 613 asthma patients, reported that 33% of these patients were wrongly diagnosed [23]. It is critical to address the perceived low importance of spirometry, both for diagnosis and monitoring, since it may also lead to under-recognition of asthma as a diagnosis, overestimation of asthma control, as well as misdiagnoses when asthma-like symptoms are observed. Ultimately, this could lead to delayed referrals to specialists or unnecessary emergency admissions due to the non-identification of unresponsive or uncontrolled patients in a timely manner [11].

For improved long-term management of asthma patients, and to increase the knowledge of best practices and the perceived importance of spirometry, GPs, specialists, as well as allied healthcare providers (such as CRE and nurses) must become better familiarized with asthma guidelines. The development of clear and concise guidelines (especially those pertaining to asthma control), followed by a promotion of their use, value, and inclusion into medical education curricula, would help establish a working integration of those documents into practice.

Perceived contextual and systemic barriers to optimal asthma care were reported in this study. Lack of access to objective lung function test and to specific medications due to cost and lack of reimbursement from insurance, is likely hindering adherence to best practices and treatment recommendations. Providing better access to spirometry and medications in primary care settings, especially in communities where specialized centers and specialists are not readily available, appears essential to improve the current state of asthma care in Canada. It is possible that the difficulty accessing spirometry led family physicians to make alternative clinical decisions to rapidly relieve patients from asthma symptoms. Initiating a medication trial based on a suspected, yet unconfirmed diagnosis is an example of current practice, reported in this study by both GP/FPs and specialists that may be perceived as the only way to proceed in the absence of objective lung function tests, even if not aligned with current best practices [24]. Therefore, contextual and systemic barriers have direct influence on family physicians’ practice habits and clinical decision-making. To verify the validity of this perception, or to identify the factors that could explain this perception, future research could examine family physicians’ access to objective lung function tests in a community setting.

Findings from this study confirmed that the lack of patient adherence to treatment plans is perceived by healthcare providers as an important barrier to provide optimal asthma care and that there is a need to educate patients regarding recognition of symptoms, better adherence, complacency issues, and timely intervention. This is supported by a survey which has demonstrated that a high proportion of patients (97%) believed their asthma to be controlled, when according to guidelines, it would have been considered uncontrolled for 47% of them (4). This is also supported by the observation that patients often prefer living with slightly more severe symptoms, in order to reduce their medication intake [25].

Results of this study show that healthcare providers do not see the value of basic recommendations such as providing written action plan or individualizing type of device, which could suggest complacency or lack of education regarding their importance. Healthcare providers are likely overestimating the patient capacity to retain and follow a verbal plan. Despite the benefits of written action plans repeatedly reported in literature and guidelines, their underuse combined with inappropriate follow-ups to assess asthma control, contribute to a lack of patient adherence to their treatment plan [26–31]. Patients and healthcare providers often do not perceive asthma as a potentially life-threatening condition, despite the fact that 250 Canadians die from asthma complications each year [32]. This highlights a need to raise awareness among healthcare providers regarding consequences of poor asthma control, and to enhance the perceived value of using written as opposed to verbal action plans.

It has been demonstrated that prevention and control of chronic conditions is optimized by the presence of a multidisciplinary, collaborative care team, involving physicians and allied health-care providers [33]. Findings from this study indicated that there is confusion regarding the individual roles and responsibilities of the physician, the nurse, the CRE and the pharmacist. Each of these healthcare providers could play a specific role in the correction of these identified gaps.

A first step towards optimal multidisciplinary management of asthma would be to provide clear guidelines about “who does what and when”. This would have to be applied to the individual community contexts since specialized personnel such as CREs may not be readily accessible leading to greater involvement by pharmacists and nurses. Previous studies suggested that pharmacists should routinely verify proper patient use of their device and provide education regarding medication and the patient treatment plan [34]. A recent survey among Canadian physicians reported that most practitioners were in favour of increasing pharmacists’ involvement related to management of asthma patients [35]. A survey distributed among Canadian community pharmacists in 2006 revealed that less than 10% of those surveyed had assessed if their clients used their asthma devices properly [36]. It is known that patients who incorrectly use their devices are more likely to have poor asthma control, and visit emergency departments more frequently [37]. The pharmacist’s role has already evolved to include this task in various healthcare locations and settings; however further research is needed to determine the educational needs of the pharmacists, as well as the best way to communicate and collaborate with physicians (e.g. reporting on compliance) and to increase their current scope of practice.

Government recognition and support for policies that would increase access to CREs, either through centralized asthma clinics or itinerant CRE, could certainly contribute to optimizing patient care as well as physician time and efficiency. Another potential solution to help achieve a clearer understanding of roles and responsibilities in multidisciplinary care would be to provide opportunities, within community practice settings, for interprofessional continuing education (IPCE) or interprofessional continuing development (IPCD) [38, 39]. However, evidence indicates that these programs have had little to no significant impact on professional practice [40]. It would therefore be important that future development of educational activities in asthma be designed around evidenced based needs, such as those identified by this needs assessment study.

### Study limitations

Given the objectives of this needs assessment, the focus of this manuscript was on areas of improvement that could be targeted by educational interventions. Therefore, areas where care was reported to be optimal were not included. As all self-reported studies, there is the possibility of erroneous self-assessment bias. Participation of healthcare providers was voluntary, which could introduce a selection bias. To mitigate potential bias, a purposive sampling, including multiple stakeholders having different years of practice was implemented to increase how representative the sample is in relation to the healthcare population working in community settings. Small sample sizes and skewed distribution in response to certain items did not allow for valid Chi square tests to assess differences between sub-groups; therefore, differences observed through valid Chi square tests only were reported. This study was conducted among community settings in the 4 largest Canadian provinces only. Respective roles and responsibilities of healthcare providers might differ in community settings of smaller provinces and therefore, findings should be generalized with caution.

## Conclusion

This Canadian needs assessment identified gaps and challenges in the treatment and management of adults with asthma using the perspective of multiple stakeholders involved in asthma care. This study also reports many gaps that were identified more than a decade ago, but that are still currently present in community practice settings, despite several attempts and strategies to overcome them. Most importantly, this study leads to a better understanding of the specific causes that could explain the observed challenges and needs of healthcare providers and patients. Many of the deficiencies pertained to lack of knowledge, confidence, and skills to properly perform specific tasks for optimal treatment and management of asthma, which could be addressed through educational activities. Future continuing medical education needs to be adapted to the needs it aims to achieve (e.g. knowledge vs. skills gaps) to obtain concrete improvement in practice. Providers in community settings also require access to adequate materials, resources (e.g. spirometry), and training to support optimal care of adult patients with asthma.

## Abbreviations

CTS: Canadian Thoracic Society
CRE: Certified Respiratory Educators
FP: family physicians
GINA: Global Initiative for Asthma
GP: general practitioners
IPCD: interprofessional continuing development
IPCE: interprofessional continuing education
SPE: specialists
SPSS: Statistical Package for the Social Sciences
